# Early Detection of Left Ventricular Systolic Dysfunction Using Two-Dimensional Speckle Tracking Strain Evaluation in Healthy Subjects after Acute Alcohol Intoxication

**DOI:** 10.1111/j.1540-8175.2012.01717.x

**Published:** 2012-05-29

**Authors:** Patricia Reant, Warren Chasseriaud, Xavier Pillois, Marina Dijos, Florence Arsac, Raymond Roudaut, Stephane Lafitte

**Affiliations:** Cardiologic Hospital, University Hospital Center of Bordeaux, CIC0005, University of BordeauxBordeaux, France

**Keywords:** binge drinking, speckle tracking strain echocardiography, alcohol intoxication

## Abstract

Objectives: We evaluated the ability of two-dimensional speckle tracking strain echocardiography to detect left ventricular (LV) systolic dysfunction as compared with LV ejection fraction (EF) in healthy subjects following acute alcohol intoxication. Methods and Results: In total, 25 healthy subjects were investigated using echocardiography 4–6 hours after the onset of alcohol intoxication at a regional festive gathering, and then compared to 23 healthy control subjects without alcohol consumption. Heart rate, blood pressure, blood alcohol level, LV volumes, EF, shortening fraction, E/A ratio, as well as global longitudinal strain (LS) were recorded. Mean blood alcohol level was 1.3 ± 0.3 g.L^−1^. Mean systolic blood pressure and heart rate were slightly increased in the alcohol group compared to controls (147.5 ± 21.8 mmHg vs 127.0 ± 9.9 mmHg, P = 0.003, and 79.7 ± 10.7 bpm vs 70.6 ± 7.6 bpm, P < 0.001, respectively). While there was no significant difference in terms of LVEF (62.9 ± 4.4% vs 64.8 ± 5.9%, P = 0.18) or shortening fraction (34.7 ± 5.9% vs 36.0 ± 4.3%, P = 0.54), global LS was significantly impaired (–17.8 ± 2.0% vs −21.2 ± 1.8%, P < 0.001). In addition, subjects who consumed alcohol had increased LV end-diastolic (108.3 ± 20.1 mL vs 95.5 ± 14.6 mL, P = 0.037) and end-systolic volumes (41.6 ± 11.4 mL vs 33.7 ± 6.9 mL, P = 0.024), along with depressed aortic time-velocity integral (19.9 ± 3.2 mL vs 21.9 ± 2.5 mL, P = 0.034). According to multivariate linear regression analyses, blood alcohol level was the only factor significantly associated with global LS (β=−3.6 ± 1.0, P = 0.005). Conclusion: Alcohol intoxication around festive days induces acute LV contraction abnormalities, which may be detected using global LS by speckle tracking at an earlier stage and more accurately than LVEF decreases.

Previous studies have reported a depression in left ventricular (LV) contractility after acute ethanol consumption based on invasive hemodynamic approaches,^1^ systolic time-interval evaluation (preejection period/LV ejection time),^2^,^3^ or ejection fraction (EF) by echocardiography.^4^ In studies on animals and healthy subjects, alcohol intoxication was shown to cause a dose-dependent impairment of cardiac contractility,^5^,^6^ with systemic inflammatory responses being considered as one of the pathophysiological mechanisms. A recent clinical magnetic resonance imaging (MRI) study reported a reversible myocardial injury with myocardial hyperenhancement after binge drinking, but without any decrease in LV systolic function.^7^

Myocardial deformation analysis using two-dimensional (2D) speckle tracking echocardiography has already been validated, showing high levels of reproducibility and accuracy in detecting subtle early LV systolic function abnormalities (depressed longitudinal deformation) in heart disorder patients without an altered EF.^8^–^14^

Therefore, our study aimed to investigate the ability of 2D speckle tracking strain echocardiography to detect early LV systolic dysfunction as compared with conventional LVEF evaluation in healthy subjects after acute alcohol intoxication versus a control group of healthy nonintoxicated subjects.

## Methods

### Study Protocol

During a regional festive gathering, consecutive healthy men subjects admitted to the aid station were consecutively evaluated by the same observer using echocardiography, 3 to 4 hours after the onset of acute alcohol intoxication.

A control group of volunteers, comprising medical students at our University Cardiologic Hospital, was recruited, given the condition that they had not consumed any alcohol for at least 10 days prior to the study.

To be eligible as healthy subjects, subjects had to be free from any known cardiovascular disease, diabetes, and hypertension.

The study excluded subjects presenting insufficient ultrasound image quality, defined as more than three LV segments being suboptimally visualized by conventional echocardiography.

Echocardiographic measurements were performed by a second observer, blinded to the subjects’ levels of alcohol consumption.

Systolic and diastolic blood pressures, heart rate, and blood alcohol level were systematically measured immediately prior to the echocardiography.

In order to estimate the blood alcohol levels, a portable breathalyzer approved by the National Authorities was used, with measurements being repeated three times and their mean values determined.

Written informed consent was obtained from all subjects. In accordance with the local ethical guidelines, this observational study was designed to be performed in the context of popular festivities.

### Transthoracic Echocardiographic Data Acquisitions

The subjects were examined in the left lateral decubitus position using a Vivid Q commercial ultrasound scanner (General Electric Healthcare, Haifa, Israel) with phased-array transducers (M4S-RS).

Thereafter, 2D data acquisitions were obtained, including parasternal long- and short-axis views and three standard apical views. For each view, three consecutive cardiac cycles were recorded during quiet respiration. Grey-scale recordings were optimized for LV evaluation at a mean frame rate of at least 50 sec^−1^. Color Doppler recordings were obtained in order to exclude valvular dysfunction, and Doppler flow recordings were performed with a horizontal sweep velocity of 100 mm/sec.

### Echocardiographic Analysis

All standard measurements were taken by the same observer using dedicated software (EchoPac PC, version BT11; General Electric Medical Systems, Horten, Norway), including percentage of LV fractional shortening, LV outflow tract time-velocity integral, peak velocity of E- and A- waves of the mitral inflow, as well as tissue Doppler analysis of max velocity E’ peak at the lateral mitral annulus. E/E’ ratio was calculated to represent LV end-diastolic pressure. Based on apical four- and two-chamber views, LV volumes and EF were measured according to Simpson's rule. All measurements were made by averaging three cardiac cycles.

For longitudinal strain (LS) analysis, the software automatically tracked the contour on subsequent frames after three endocardial markers were placed in an end-diastolic frame. Adequate tracking could be verified in real time and corrected by adjusting the region of interest or manually correcting the contour so as to ensure optimal tracking. In addition, LS was assessed in apical views. Average LS was calculated for the 17 segments in relation to the strain magnitude at aortic valve closure. Longitudinal systolic deformation was characterized as shortening, and using the strain definition, systolic indices yielded a negative value.

### Reproducibility

The second observer performed all the echocardiographic measurements, which were later repeated blindly for each parameter. The data were also analyzed by a third blinded observer for 10 of the subjects. Intraobserver reproducibility was calculated using the average difference between 10 measurements, and interobserver reproducibility was assessed as the absolute difference divided by the average of the two observations for all parameters.

### Statistical Analysis

All statistical analyses were performed using SPSS for Windows (version 15.0, SPSS Inc., Chicago, IL, USA). All data were expressed as mean ± standard deviation.

Between-group comparisons were made using paired *t*-tests or the Mann-Whitney test where necessary. A P-value of <0.05 was considered statistically significant. Univariate and multivariate linear regression analyses were performed in order to identify potential links with global LS. The following independent variables were examined: systolic and diastolic blood pressure, heart rate, blood alcohol level, LV shortening fraction, biplane LV end-diastolic volume, biplane LVEF, aortic time-velocity integral, and E/E’.

## Results

### Study Population

Among the 25 screened subjects with acute alcohol intoxication, two were excluded from the analysis on account of having at least nonvisualized three LV segments, considered to be of insufficient image quality. The healthy controls without alcohol consumption consisted of 23 subjects.

The population participants who consumed alcohol were all men, with a mean age of 25 ± 8 years. Mean blood alcohol level was of 1.3 ± 0.3 g.L^−1^. Mean systolic blood pressure and heart rate were slightly higher in the alcohol group compared to controls, with the differences being statistically significant (147.5 ± 21.8 mmHg vs 127.0 ± 9.9 mmHg, P = 0.003, and 79.7 ± 10.7 bpm vs 70.6 ± 7.6 bpm, P < 0.001, respectively). Clinical characteristics of the two study groups are summarized in Table I.

**TABLE I:** Clinical and Echocardiographic Characteristics of the Population

	Subjects with Alcohol Intoxication (n = 23)	Control Subjects (n = 23)	P
Men (%)	100	100	1
Age (yrs)	24.8 ± 8.1	27.0 ± 9.2	0.44
Systolic blood pressure (mmHg)	147.5 ± 21.8	127.0 ± 9.9	0.003
Diastolic blood pressure (mmHg)	88.3 ± 17.1	79.7 ± 8.1	0.05
Heart rate (bpm)	79.7 ± 10.7	70.6 ± 7.6	<0.001
LV end-diastolic diameter (mm)	49.9 ± 4.0	49.7 ± 2.6	0.55
LV end-systolic diameter (mm)	32.3 ± 3.5	31.8 ± 2.7	0.55
LV shortening fraction (%)	34.7 ± 5.9	36.0 ± 4.3	0.54
Biplane LV end-diastolic volume (mL)	108.3 ± 20.1	95.5 ± 14.6	0.037
Biplane LV end-systolic volume (mL)	41.6 ± 11.4	33.7 ± 6.9	0.024
Biplane LVEF (%)	62.9 ± 4.4	64.8 ± 5.9	0.18
Global longitudinal strain (%)	–17.8 ± 2.0	–21.2 ± 1.8	<0.001
Aortic velocity time integral (cm)	19.9 ± 3.2	21.9 ± 2.5	0.034
E/A ratio	1.6 ± 0.3	1.7 ± 0.6	0.44
E/E’	4.7 ± 1.4	5.8 ± 1.3	0.10

### Echocardiographic Measurements

Echocardiographic characteristics of the study population are summarized in Table I. There was no significant difference between the subjects who did and did not consume alcohol in terms of LVEF (62.9 ± 4.4% vs. 64.8 ± 5.9%, P = 0.18) or LV shortening fraction (34.7 ± 5.9% vs. 36.0 ± 4.3%, P = 0.54).

However, global LS was significantly lower in subjects with alcohol intoxication (–17.8 ± 2.0% vs −21.2 ± 1.8%, P < 0.001), larger LV volumes (P < 0.05), and lower aortic time-velocity integral. There was no linear relation between global LS and LV volumes or blood pressure, nor was there any correlation between blood alcohol levels and clinical or other echocardiographic parameters.

### Reproducibility

Mean intra- and interobserver variability of global LS was 7% and 8%, respectively.

### Regression Analyses

The results of univariate analysis (P-values) for the different parameters and their relation to global LS are provided in Table II. Systolic blood pressure, blood alcohol level, LV shortening fraction, end-diastolic and end-systolic LV volumes, EF, and time-velocity integral were significantly linked to global LS.

**TABLE II:** Analyses of Association with Altered Global Longitudinal Strain

	Univariate		Multivariate	
		
Variables	βn ± SE	P-Value	βn ± SE	P-Value
Systolic blood pressure	–0.0583 ± 0.0260	0.03	0.0009 ± 0.0322	0.98
Diastolic blood pressure	–0.0545 ± 0.0387	0.17	0.0996 ± 0.0608	0.13
Heart rate	–0.0697 ± 0.0367	0.06	–0.0118 ± 0.037	0.75
Blood alcohol level	–3.318 ± 0.466	<0.0001	–3.628 ± 1.003	0.005
LV shortening fraction	15.644 ± 6.884	0.03	–2.432 ± 9.113	0.79
Biplane LV end-diastolic volume	–0.0793 ± 0.0146	<0.0001	–0.016 ± 0.0147	0.32
Biplane LVEF	22.513 ± 9.143	0.02	14.633 ± 8.547	0.12
Aortic time-velocity integral	0.348 ± 0.1024	0.002	–0.0565 ± 0.1099	0.62
E/E’	0.678 ± 0.399	0.10	–0.1675 ± 0.5568	0.77

In a multivariate analysis, regarding the separate contribution to global LS of each parameter among all parameters selected, only alcohol intoxication was found to significantly and inversely contribute to global LS (β=−3.6 ± 1.0, P = 0.005) (Table II).

**Figure 1:**
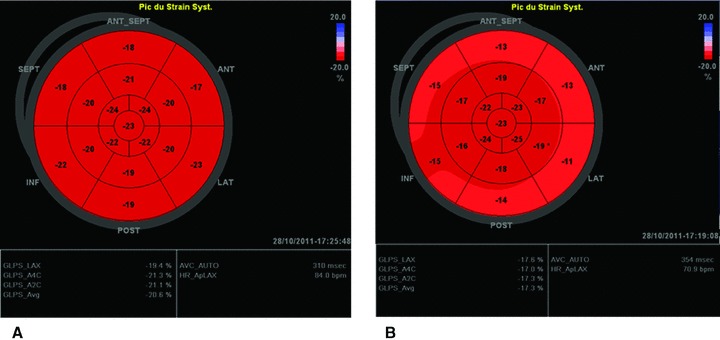
Example of longitudinal strain in healthy subjects without **A.** and with **B.** alcohol intoxication. **A.** Global longitudinal strain in a healthy control subject without alcohol consumption, and **B.** global longitudinal strain in a healthy subject with alcohol consumption.

Figure 1 shows an example of GLS analysis in a control subject (A) and intoxicated subject (B).

## Discussion

In this study, our main finding was that myocardial LS analysis using 2D speckle-tracking echocardiography allowed us to detect early LV systolic dysfunction immediately after acute alcohol intoxication in men, whereas LVEF and LV shortening fraction did not differ from those of control healthy subjects.

### Capacity of Global LS to Detect LV Dysfunction Earlier than LVEF and Shortening Fraction in Different Pathophysiological Situations

The conventional evaluation of LV systolic function by means of shortening fraction or EF is limited by the percentage variability of measurements (10–15%). The assessment of LV longitudinal deformation using 2D speckle tracking has been largely validated against sonomicrometry and MRI as reference methods.^8^,^9^ Speckle tracking allows for a quick and easy quantification (<1 min), with greater reproducibility (7% intraobserver variability in our experience) than LVEF or shortening fraction.

Moreover, global LS analysis, also known as automated function imaging, permits the exploration of the myocardial pathophysiology in new ways, particularly by detecting early alterations in the longitudinal component of LV systolic function, while the radial component is considered normal.^10^–^12^ These findings are observed with normal ageing as well as with the typical precursors of heart failure with a normal EF, as seen in hypertension, diabetes, hypertrophic cardiomyopathy, severe aortic stenosis, and ischemia.^10^–^15^ In hypertensive patients, interstitial and perivascular fibrosis is likely to affect primarily the subendocardium. Longitudinal fibers, as a consequence of their prominent subendocardial location, are more vulnerable to fibrosis and hemodynamic overload. Thus, subendocardial long-axis function may be impaired long before circular fiber dysfunction develops in the midwall or radial fiber dysfunction in the subepicardial layers. Consequently, subendocardial long-axis function is viewed as a potential marker of subclinical LV dysfunction in several disease conditions.^13^,^14^ Similarly, longitudinal fibers are more susceptible to ischemia than radial fibers. Thus, there appears to be an entire spectrum of systolic function abnormalities, ranging from normal systolic heart function to systolic heart failure, with heart failure with normal EF being located in between.

### Effects of Acute Alcohol Intoxication on LV Function

In studies on animals and healthy subjects, alcohol intoxication was shown to cause a dose-dependent impairment of cardiac contractility,^5^,^6^ with systemic inflammatory responses being considered as one of the underlying pathophysiological mechanisms.^16^ Ethanol may modify cell activation by specific interactions with cell membrane molecules, thereby involving the innate immune system.^16^

Several published studies reported a depression in LV contractility following acute ethanol consumption based on invasive hemodynamic approaches,^1^ systolic time-interval evaluation (preejection period/LV ejection time),^2^,^3^ or EF by echocardiography.^4^

Delgado et al. investigated the effects of oral doses of whiskey on LV function in a group of normal volunteers (n = 13).^4^ At 30 min after ingesting the alcohol, the heart rate was increased by 11%, while the fractional change across the minor axis of the LV decreased by 6% and LVEF by 4% (P < 0.001). These results, however, have to be interpreted with caution, given the small sample size and considering that LVEF measurements exhibit 10–15% variability, particularly when using echocardiographic systems from the mid-1970s. By contrast, variability of global LS measurements approximated 7%, as based on our own experience.

More recently, a clinical MRI study reported a reversible myocardial injury as defined by myocardial hyperhancement after binge drinking, but without any concomitant decrease in LV systolic function.^7^ This latter study demonstrated that alcohol had no significant effect on LVEF immediately after acute alcohol intoxication, in line with our own observation. Consequently, radial function may be preserved in the initial phase, whereas the longitudinal component could possibly be altered at an earlier stage, which is mainly accounted for by the greater radius of curvature of longitudinal fibers as compared to radial fibers (Laplace's law). Due to the greater radius curvature, the longitudinal myofibers are deemed more susceptible to the different phenomena altering LV contractility compared to radial fibers.

### Limitations

LVEF was evaluated in 2D using biplane Simpson's method, although 3D volume quantification might have been more accurate for quantifying EF and volumes. As only male subjects were included in our study, the effects of alcohol intoxication on LV systolic function in women were not assessed. In this study, we did not perform serial echocardiography on the alcohol intoxicated subjects. This should have strengthened our results.

Preload and afterload modifications may exert an impact on LV strains. In severe aortic stenosis or hypertensive cardiopathy, afterload elevation was shown to influence more specifically the longitudinal component of LV systolic function, due to the greater radius of curvature of longitudinal fibers. Soon after alcohol intoxication in healthy subjects, blood pressure, heart rate, and volumes were shown to be increased. We, therefore, assumed that preload and afterload elevations may have an impact on global LS. However, multivariate regression analyses demonstrated that blood alcohol levels alone were significantly related to global LS, whereas blood pressure, heart rate, LVEF, and LV volumes were not.

## Conclusions

Global LS by speckle tracking echocardiography allows for a more accurate detection of early LV systolic dysfunction after festive alcohol intoxication compared to LVEF, with the results being independent from blood pressure, heart rate, and LV volumes variations.
